# Moderating Effects of Age and Gender on the Relationship Between Pediatric Obsessive–Compulsive Symptoms and Parental Accommodation

**DOI:** 10.1007/s10578-025-01816-4

**Published:** 2025-02-26

**Authors:** Megan C. DuBois, Evan Realbuto, Christopher A. Flessner

**Affiliations:** Department of Psychological Sciences, Kent State University, Kent, OH USA

**Keywords:** Pediatric obsessive–compulsive symptoms, Parental accommodation

## Abstract

Parental accommodation is a well-established anxiogenic parenting practice that is ubiquitous among parents of youth with obsessive–compulsive symptoms (OCS). Accommodation is associated with heightened symptom severity (i.e., high levels of accommodation reinforce and maintain OCS). The present study sought to evaluate whether child age and gender moderated the relationship between parental accommodation and symptom severity. Participants included parents of children with a broad range of psychiatric disorders, as well as some youth with no psychiatric disorder (N = 61, children ages 7–17). Parents completed questionnaires related to their accommodation practices and their child’s obsessive–compulsive symptoms. Age significantly moderated the relationship between accommodation and symptom severity, such that the relationship was stronger among older children. Gender significantly moderated the relationship between accommodation and symptom severity, such that the relationship was stronger among boys. Additional research is needed to further delineate the impact of age and gender on parental accommodation and OCS.

## Introduction

Obsessive–compulsive disorder (OCD) is a chronic and impairing psychiatric disorder that affects up to 2–3% of children and adolescents [51]. OCD is characterized by both obsessions (i.e., intrusive, persistent, and unwanted thoughts or images) and compulsions (i.e., ritualized behaviors or mental acts performed to alleviate distress associated with obsessions; American Psychiatric Association [APA], 2022). Age and sex/gender are thought to play important roles in the onset and expression of OCD. For example, evidence supports an earlier onset of OCD symptoms in males, but a higher prevalence of adult women with clinically impairing OCD [26]. Different types of obsessions, compulsions, and comorbidity profiles exist across males and females [5, 26]. There may also be phenotypic differences in childhood- and adult-onset OCD, though research as to why this occurs or how it affects long-term outcomes is mixed [16, 35]. Age and sex/gender may drive heterogeneity of OCD symptom presentation. The aim of the current paper is to evaluate age and gender identity as potential factors that impact pediatric OCD symptom severity.

Across age and gender, compulsions and other obsessive–compulsive symptoms (OCS) and related behaviors differ from developmentally appropriate rituals or mental acts (e.g., daily morning and bedtime routines). These behaviors are time-consuming, inflexible, and, in conjunction with obsessions, contribute to significant perceived distress and place a substantial burden on parents and the family system [33, 38]. Moreover, families are not only impacted by a child’s OCS, but several parent-level variables (e.g., family dynamic, parent psychopathology, parenting practices) influence symptom expression and severity [13, 14, 27, 50]. This bidirectional relationship between parent(s) and child highlights the need to better understand contextual factors that affect pediatric OCS within the family system.

Parental accommodation (i.e., parents modifying their behavior in response to their child’s anxiety or OCS) is a well-established, bidirectional anxiogenic parenting practice that influences and is influenced by youth OCD [23, 34]. High levels of accommodation are associated with increased symptom severity [15, 47]. Roughly 70% of parents report daily accommodation, and up to 99% of parents reporting some accommodation weekly [13, 14, 34]. Accommodation can take many forms (e.g., allowing a child to be late for school to finish a morning routine compulsion, excessively washing food before mealtimes, cooking multiple meals, etc.) parents who accommodate their child’s OCS often do so with the intention of alleviating unwanted symptoms. Accommodation can reduce a child’s short-term distress, however, this ultimately maintains OCS long-term by facilitating avoidance and other maladaptive coping strategies [22, 39].

To date, studies investigating the relationship between accommodation and OCS are largely cross-sectional. However, some research suggests that parental accommodation is a strong predictor of OCS severity two years later [15]. Moreover, reductions in accommodation in OCD treatment studies are associated with decreased symptom severity post-treatment, indicating a persistent relationship that extends beyond one point in time [22]. For example, in a large sample of youth with OCD, reductions in parental accommodation during a 10-session CBT program mediated the relationship between OCD severity and parent-rated impairment, suggesting that interventions aimed at the identification and reduction of accommodation (e.g., CBT) may drive a prevailing reduction in impairment regardless [21]. Collectively, these findings, and their potential implications in treatment, emphasize the importance of better understanding factors that precipitate (i.e., predict) parental accommodation as well as influence accommodation’s effect on symptom severity.

At present, the majority of research has only explored clinical correlates and predictors of parental accommodation (i.e., what factors influence accommodation)*.* In a large systematic review, only OCS severity was a significant correlate or predictor in all studies examined [42]. Furthermore, four of seven studies in Watson and colleagues’ review (e.g., [7, 13, 14, 29, 45]) found that child oppositional or externalizing behavior was associated with higher levels of parental accommodation. The review authors noted that, despite these consistencies, a lack of conceptual and methodological congruence across studies has prevented the advancement of any reliable conclusions regarding which clinical correlates, if any, are most pertinent to understanding parental accommodation. Watson and colleagues [42] argued that, rather than identifying new predictors of accommodation, future research could benefit from further examination of what we already know about accommodation.

The current study seeks to address this gap in the literature by examining factors that influence (i.e., moderate) the relationship between parental accommodation and OCS severity (i.e., accommodation as the predictor rather than outcome). Because accommodation occurs in nearly all families of children exhibiting some OCS, it may be particularly beneficial to explore factors that strengthen this relationship once it is already present in the parent–child dynamic. To date, only one such study has pursued this line of inquiry by examining child-level variables as moderators [46]. In this clinical sample of youth and their caregivers, comorbid anxiety disorders (but not comorbid mood, oppositional, or attention-deficit/hyperactivity disorders) moderated the relationship between OCS severity and parental accommodation, such that *not* having a comorbid anxiety disorder indicated a stronger positive relationship.

No empirical study to date has specifically examined age or gender as moderators of the accommodation and OCS relationship. Both age and gender may account for heterogeneity of symptom presentation and trajectory of illness [16, 26], and thus are worthwhile variables to explore. In a meta-analysis of the relationship between family accommodation and OCS, Wu and colleagues [47] compared effect sizes between pediatric OCD studies and adult OCD studies to evaluate differential effects of age. However, this did not include age-related differences within pediatric populations (e.g., child versus adolescent). Some work broadly supports the idea that more accommodation occurs in younger children [34]. From a developmental perspective, this makes sense given that younger children’s routines are more closely linked with their parents than adolescent’s routines.

There is currently no clear pattern between accommodation and gender independently [47]. Some research suggests that, beyond OCS and accommodation literature, certain parenting practices influence anxiety differently between boys and girls [40, 49]. For example, in a large cross-sectional study that examined the roles of parenting practices on later anxiety, higher parental control had a greater negative impact on girls’ anxiety symptoms than it did on boys’ [3]. Similarly, girls are also more susceptible than boys to increases in their own anxiety during adolescence as a function of their parents’ heightened anxiety when they are young children [31]. There is substantially less research highlighting how parenting influences OCS differentially between boys and girls, but given the overlap of OCD and anxiety disorders, it is possible that a similar effect may exist in OCS.

Ultimately, parental accommodation in pediatric OCS is ubiquitous, and its influence on symptom severity is well established in cross-sectional research [23, 34]. Some OCS literature supports age (e.g., different disorder trajectories depending on child versus adult onset [16]) and gender (e.g., different symptom presentation across genders [26], differences in symptom onset and course. There is a significant gap in research that examines child-level variables impacting the OCS/accommodation relationship. Taken together, a logical first step in this work is to look at whether older versus younger children, and boys versus girls, have different responses to parental accommodation. Thus, the aim of the current study is to determine whether (1) age or (2) gender moderate the relationship between parental accommodation and OCS severity. We expect that there will be a stronger relationship between accommodation and symptom severity in older children than in younger children, in part due to parental involvement in their child’s behavior(s) being more normative among younger children and therefore exerting a less potent influence on symptom severity. We also expect that there will be a stronger relationship between accommodation and symptom severity in girls than in boys, given broader anxiety research that suggests facets of parenting related to accommodation (e.g., control) may more strongly impact female anxiety.

## Methods

### Participants

Youth and their caregivers were recruited as part of a larger study examining parenting practices and cognitive functioning in pediatric anxiety and related problems (e.g., OCS). Participants self-identified from flyers and website advertisements posted throughout Northeast Ohio. Children were included in the study if they were 7 through 17 years old. Though anxiety disorders were the focus of the larger study, no diagnosis was required to participate, and thus the study involved a mixed clinical/non-clinical sample (for similar mixed study populations in parental accommodation research, see [11]). Child exclusion criteria included (1) a diagnosis of major depressive disorder (assessed via structured diagnostic interview) and (2) a diagnosis of autism spectrum disorder (determined via parent-report). In total, 61 parents or guardians completed the Family Accommodation Scale—Anxiety (FASA), the Spence Children’s Anxiety Scale Parent Report–Obsessive–Compulsive Subscale (SCAS-P), and the Child Behavior Checklist (CBCL). Caregivers’ ages ranged from 31 to 67 (M = 44.25, SD = 7.0), and 90% of informants were female (i.e., child’s biological, adoptive, or foster mother). Children’s ages ranged from 7 to 17 (M = 11.92, SD = 2.3) and were 62% female. The majority of families were White/Caucasian, non-Hispanic/Latino, and had an annual household income greater than $60,001. No participants identified as transgender or nonbinary. See Tables 1 and 2 for complete demographic information, including parent/child race and ethnicity breakdown and child diagnostic information.

**Table 1:** Parent & child demographics

	Parent		Child	
	*n*	*%*	*n*	*%*
*Sex*				
Female	55	90.2	38	62.3
Male	6	9.8	23	37.7
*Ethnicity*				
Hispanic/Latino	2	3.3	4	6.6
Non-Hispanic/Latino	57	94.4	56	91.8
Missing	2	3.3	1	1.6
*Race*				
White/Caucasian	58	95.1	53	86.9
African American	2	3.3	4	6.6
Asian	0	0	1	1.6
Other	1	1.6	3	4.9
*Annual Household Income*				
< $10,000	4	6.6	–	–
$10,000-$50,000	11	18.0	–	–
$50,001-$60,000	8	13.1	–	–
> $60,001	37	60.7	–	–
Missing	1	1.6	–	–

*N* = 61. Parents were on average 44.3 years old (*SD* = 7.0) and children were on average 11.9 years old (SD = 2.3)

**Table 2:** Child Diagnostic information

	Diagnostic Information^a^	
	*n*	*%*
Panic Disorder	0	0
Agoraphobia	0	0
Obsessive–Compulsive Disorder	12	19.7
Post-Traumatic Stress Disorder	2	3.3
Generalized Anxiety Disorder	23	37.7
Separation Anxiety Disorder	3	4.9
Social Anxiety	20	32.8
Specific Phobia^b^	27	44.3
Tic Disorder^c^	5	8.2
ADHD^d^	7	11.5
Trichotillomania^e^	6	9.8
Oppositional Defiance Disorder	3	4.9
No Psychiatric Diagnosis^f^	19	33.3

^a^Child diagnoses obtained from Anxiety and Related Disorders Interview Schedule (ADIS)

^b^Includes animal, natural environment, blood-injection/injury, situational, and other phobia

^c^Includes Tourette Disorder, Chronic Motor Tic Disorder, Chronic Vocal Tic Disorder, and Transient Tic Disorder NOS

^d^Includes ADHD-Inattentive Type, ADHD-Hyperactive Type, and ADHD-Combined Type

^e^As measured by the Trichotillomania Diagnostic Interview (TDI)

^f^Indicated by clinical severity score < 4 on the ADIS. Does not include subthreshold symptoms

## Procedure

The study was approved by the university’s Institutional Review Board (IRB). Interested families contacted the university clinic and research staff determined eligibility via a brief phone screen. As part of the larger study of pediatric anxiety and cognitive functioning, eligible participants were scheduled for two in-person clinic visits (Day 1 and Day 2) separated by approximately three weeks. Prior to the Day 1 appointment, families reviewed the consent form and completed a battery of self-report measures used for the purpose of the present research. During the Day 1 appointment, parental consent and child assent were formally obtained and diagnoses were confirmed via a structured diagnostic interview conducted by a trained graduate level research assistant (see Table 2).

## Measures

### The Family Accommodation Scale—Anxiety (FASA [24];)

The FASA is a 13-item parent- and self-report measure of parental accommodation in anxiety disorders. Only the parent-report version was used for this study. Parents are asked to rate the frequency of accommodation on a 1 (Never) to 5 (Daily) point scale, with higher scores indicating more frequent accommodation. The FASA assesses parental accommodation in three domains: (1) parent participation in symptom-related behaviors (e.g., “How often did you reassure your child?”); (2) modification of functioning (e.g., “Have you modified your family routine because of your child’s symptoms?”); and (3) distress and consequences, rated on a 1–5 Likert scale ranging from No to Extreme (e.g., “Has your child become distressed when you have not provided assistance?”). The FASA is shown to have good internal consistency and convergent & discriminative validity among clinical populations [24].

The FASA was originally adapted for use from Calvocoressi and colleagues’ [6] measure of accommodation among relatives of individuals with OCD (FAS). Updated, self-report versions of the FAS for OCD remain in use (e.g., [13, 14, 30]), however, domains of accommodation measured across both the anxiety and OCD versions of the family accommodation scales are similar. A primary difference is the specific use of language such as “compulsions” or “rituals” in the FAS as opposed to more general language related to anxiety, avoidance, and reassurance in the FASA. Though OCS are the focus of the present analyses, the adapted FASA was opted for use in this larger project to capture a broader range of anxiety-related disorders in addition to OCS. Within this sample, the FASA demonstrated excellent internal consistency (α = 0.93).

### Spence Child Anxiety Scale-Parent Version (SCAS-P; Spence [37]*)*

The SCAS-P is a 38-item parent–report measure of anxiety symptoms across six domains: social phobia, separation anxiety, panic attack/agoraphobia, obsessive–compulsive disorder, generalized anxiety, and physical injury fears. Each item is rated on a 0 (Never) to 3 (Always) point scale, with higher scores indicating higher levels of anxiety. The SCAS-P has strong psychometric properties in children ages 8–12 (Spence et al., [36]) and acceptable psychometric properties in adolescents ages 13 and 14 [37]. In both age groups, the SCAS correlates strongly with established measures of child anxiety.

The obsessive–compulsive subscale was used as the primary outcome measure for this study. The subscale is composed of six questions assessing central features of OCD (e.g., “My child has to keep checking that he/she has done things right” or “My child can’t seem to get bad or silly thoughts out of their head”). SCAS-P: OCD subscale demonstrates good concurrent validity via strong correlations with other validated measures of OCD symptoms in clinical and community samples (e.g., Children’s Yale-Brown Obsessive–Compulsive Scale; [43]). Within this sample, the SCAS-P: OCD subscale demonstrated good internal consistency (α = 0.84).

### Child Behavior Checklist (CBCL [1];*)*

The CBCL is a 112-item parent-report measure assessing broad adaptive, emotional, and behavioral functioning in youth ages 6–18. Parents are asked to rate behaviors on a 0 (Never True) to 2 (Often/Very True) point scale, with high scores indicating greater frequency of emotional/behavioral problems. The anxious-depressed and inattention subscales were used in the present study to control for anxiety, depression, and attention symptoms. The anxious-depressed subscale has been identified as a useful screening tool for anxiety and affective disorders in youth [2, 9]. The CBCL inattention subscale includes multiple facets of ADHD (e.g., inattention, hyperactivity, and impulsivity, e.g., “Can’t sit still”) and discriminates youth with and without ADHD [10, 41]. Within this sample, the CBCL anxious-depressed and inattention subscales demonstrate good internal consistency (α = 0.87, α = 0.89, respectively).

### Data Analytic Plan and Preliminary Analyses

Two moderation analyses were conducted to determine whether age and gender moderate the relationship between parental accommodation and OCS severity. Child internalizing symptoms (i.e., CBCL: Anxious-Depressed subscale) and inattention (i.e., CBCL: Inattention subscale) were identified as possible covariates given higher rates of comorbidity among youth with OCD [17, 25]. Initial Pearson correlations were conducted between anxiety/depression, attention, and our primary outcome variable (SCAS-P: OCD subscale). After controlling for the number of comparisons using a Bonferroni correction, only the anxious-depressed subscale was significantly related to obsessive–compulsive symptoms (*p* < 0.001) and used as a covariate in the analyses. Basic assumptions of regression were tested prior to analyses. The SCAS-P: OCD subscale was not normally distributed. However, given our use of the PROCESS Macro that includes bootstrapping, a normal distribution is not critical, and the central limit theorem applies [12, 18].

## Results

Means and standard deviations for relevant measures are reported in Table 3.

**Table 3:** Child clinical information

	Parent-Reported Scores		
	Min	Max	M (SD)
Family accommodation scale–anxiety (FASA)	0	40	11.2 (10.2)
Spence child anxiety scale–parent (SCAS-P)			
*SCAS-P–Obsessive–compulsive subscale*	0	16	1.3 (2.6)
Child behavior checklist (CBCL)			
*CBCL–Anxious/depressed subscale*	50	89	61.3 (10.2)
*CBCL–Attention problems subscale*	50	97	58.0 (9.7)

### Age as a Moderator

**Fig. 1:**
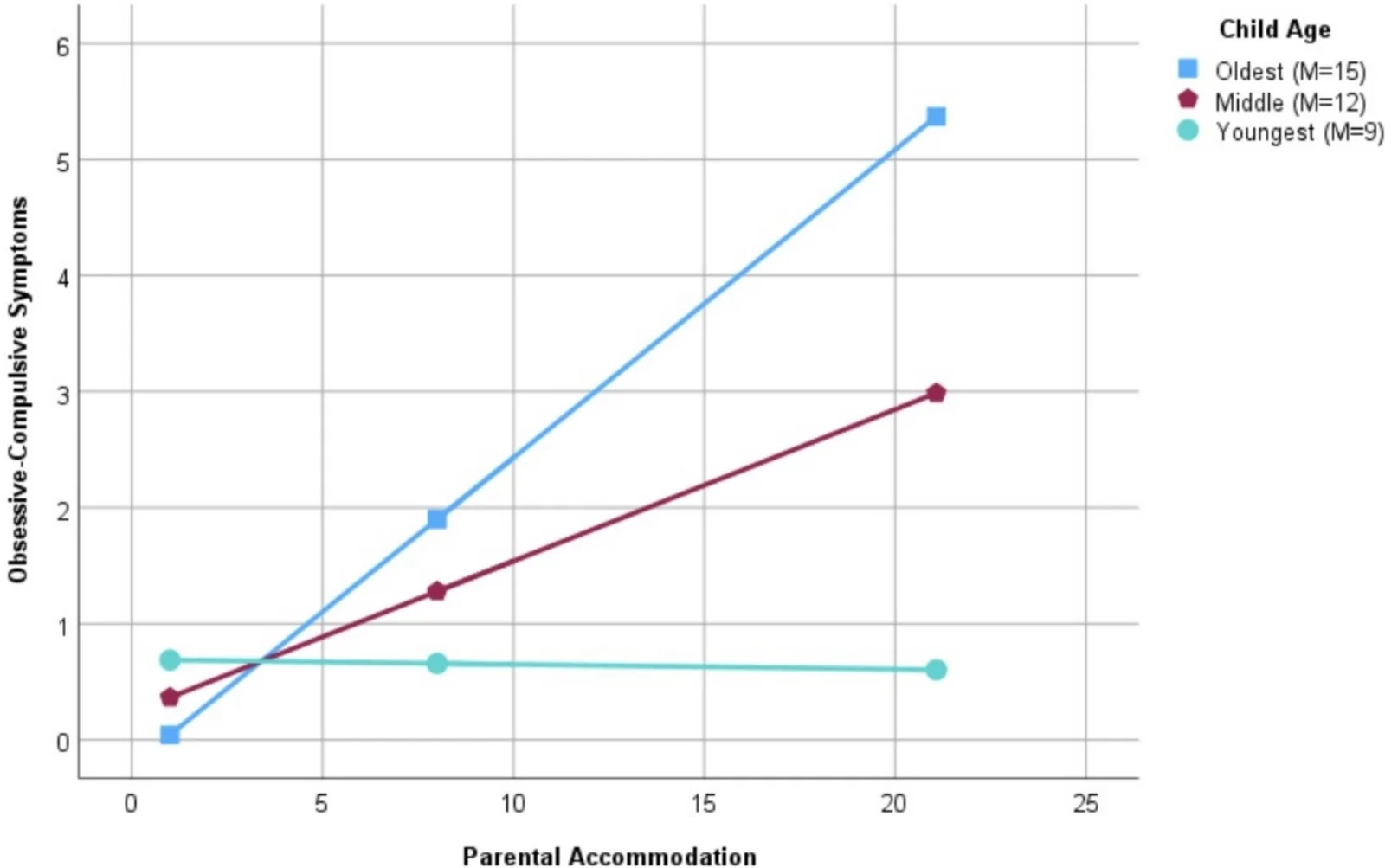
Moderation of age on the relationship between parental accommodation and obsessive–compulsive symptoms

A simple moderation analysis was conducted using SPSS’s PROCESS Macro [18] to evaluate whether child age moderated the relationship between parental accommodation and OCS, after controlling for child internalizing symptoms (i.e., CBCL: Anxious-Depressed subscale scores). The interaction between accommodation and age was significant (*b* = 0.05, *p* < 0.01), indicating that age moderated the relationship between accommodation and symptom severity (Table 4). The interaction was further probed by testing the conditional effects of accommodation at the 16th, 50th, and 84th age percentiles (Fig. 1). In middle (i.e., 50th percentile, M = 12 years of age) and older children (i.e., 84th percentile, M = 15 years of age), parental accommodation significantly predicted OCS. The relationship was stronger for the oldest group (Middle: *b* = 0.13, *p* < 0.01; Oldest: *b* = 0.27, *p* < 0.01). In younger children (i.e., 16th percentile, M = 9 years old), accommodation did not predict symptom severity (*b* = − 0.003, *p* = 0.94).

**Table 4:** Moderation analyses

	B	SE	t	p	CI
Constant	− 0.04	3.26	− 0.01	.991	[− 6.56, 6.49]
Parental Accommodation	− 0.41	0.15	− 2.79*	.007	[− 0.71, -0.12]
Child Age	− 0.15	0.17	− 0.89	.379	[− 0.49, 0.19]
Accommodation x Age	0.05	0.01	3.37*	.001	[0.02, 0.07]
Constant	− 6.06	2.56	− 2.37*	.021	[− 11.18, -0.94]
Parental Accommodation	0.26	0.09	2.79*	.007	[0.07, 0.45]
Child Gender	1.02	0.89	1.14	.256	[− 0.77, 2.81]
Accommodation x Gender	− 0.15	0.06	− 2.65*	.011	[− 0.26, -0.04]

Accommodation x Age R^2^ = .12, *p* = .001; Accommodation x Gender *R*^*2*^ = .09, *p* = .06

^*^p < .05

### Gender as a Moderator

**Fig. 2:**
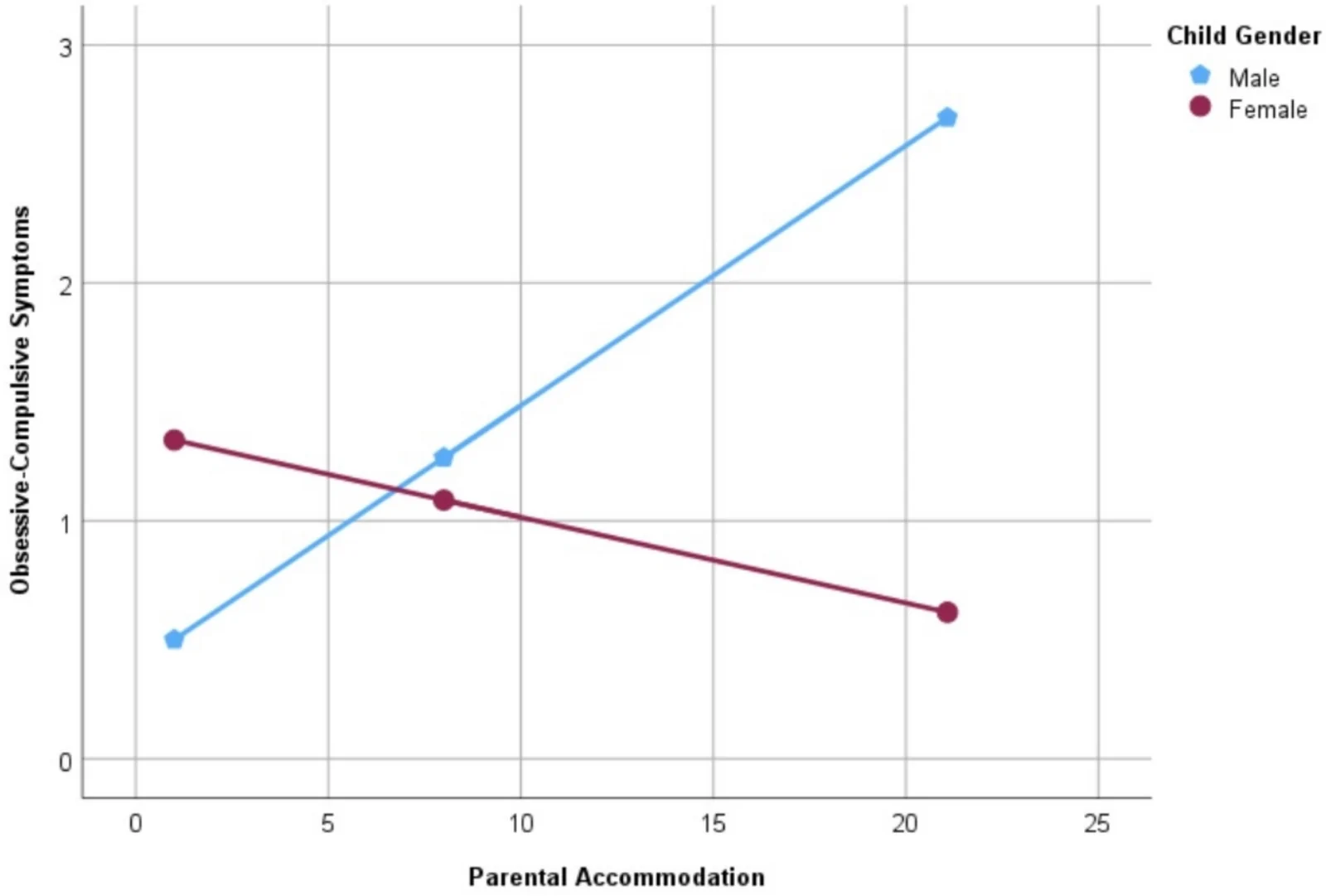
Moderation of gender on the relationship between parental accommodation and obsessive–compulsive symptoms

A simple moderation analysis was conducted to evaluate whether child gender moderated the relationship between parental accommodation and OCS, after controlling for child internalizing symptoms (i.e., CBCL: Anxious-Depressed subscale score). The interaction between accommodation and gender was significant (*b* = − 0.15, *p* = 0.01), indicating that gender moderated the relationship between accommodation and symptom severity (Table 4). The interaction was probed by testing the conditional effects of accommodation for each gender (Fig. 2). For boys, parental accommodation significantly predicted OCD symptom severity (*b* = 0.11, *p* = 0.02). For girls, accommodation did not predict symptom severity (*b* = − 0.04, *p* = 0.46).

### Exploratory Analyses

A series of exploratory post-hoc t-tests were conducted to assess whether gender differences existed with respect to individual items on the Family Accommodation Scale – Anxiety (Table 5). Parents of boys reported significantly higher scores (M = 1.4, SD = 1.2) than parents of girls (M = 0.7, SD = 1.0) on the item “Has your child become distressed when you have not provided assistance?” [*t*(58) = 2.4, *p* = 0.02]. There was trending significance on the item “Has your child become angry/abusive when you have not provided assistance?” and on the Distress and Consequences subscale total, such that parents rated higher scores for boys than for girls (*p* = 0.067 and *p* = 0.073, respectively).

**Table 5:** Gender differences in FASA items

	Male	Female	*t*(58)	*p*
	M (SD)	M (SD)		
How often did you reassure your child?	2.6 (1.2)	2.3 (1.1)	1.2	.253
How often did you provide items needed because of anxiety?	1.0 (1.1)	0.9 (1.3)	0.2	.858
How often did you participate in behaviors related to your child’s anxiety?	1.0 (1.3)	1.0 (1.3)	− 0.1	.889
How often did you assist your child in avoiding things that might make him/her more anxious?	1.1 (1.3)	0.6 (1.1)	1.5	.133
Have you avoided doing things, going places, or being with people because of your child’s anxiety?	0.6 (1.1)	0.4 (0.7)	0.9	.348
Have you modified your family routine because of your child’s symptoms?	1.0 (1.3)	0.5 (1.1)	1.5	.131
Have you had to do things that would usually be your child’s responsibility?	1.0 (1.4)	0.9 (1.3)	0.5	.609
Have you modified your work schedule because of your child’s anxiety?	1.3 (4.2)	0.2 (0.6)	1.2	.226
Have you modified your leisure activities because of your child’s anxiety?	0.4 (0.9)	0.3 (0.7)	0.9	.387
Does helping your child in these ways cause you distress?	0.9 (1.1)	0.7 (0.8)	0.8	.438
Has your child become distressed when you have not provided assistance?	1.4 (1.2)	0.7 (1.0)	2.4*	.021
Has your child become angry/abusive when you have not provided assistance?	1.0 (1.2)	0.4 (0.9)	1.9	.067
Has your child’s anxiety been worse when you have not provided assistance?	1.2 (1.2)	0.8 (1.0)	1.7	.096
FASA–Participation subscale total	6.0 (4.3)	5.4 (4.4)	0.5	.618
FASA–Modification of functioning subscale total	3.0 (4.2)	2.0 (2.8)	1.1	.295
FASA–Distress and consequences subscale total	4.4 (4.1)	2.6 (3.2)	1.8	.073

^*^p < .05

## Discussion

The aim of the present study was to evaluate age and gender as factors that influence the relationship between parental accommodation and OCS severity. Results indicated a significant moderating effect for both age and gender. In line with our hypothesis, age moderated the relationship between parental accommodation and OCS, such that higher levels of accommodation predicted more severe OCS in older children but not in younger children. Interestingly, gender also moderated the relationship between parental accommodation and OCS, but the direction of this relationship was not in line with our hypothesis. Higher levels of accommodation predicted more severe OCS in boys but not in girls.

### Age as a Moderator

Results from our first set of analyses supported our hypothesis that parental accommodation may have a larger impact on adolescents’ OCS than on younger children’s OCS. Previously, researchers have noted that accommodation is more likely to occur in younger children [34],however, whether age-related differences exist regarding the relationship between accommodation and symptom severity has largely been unexplored within the OCS and parental accommodation literature. Though results are exploratory given the cross-sectional study design, it is possible that the relationship between parental accommodation and OCS mirror a relationship that occurs between the related concepts of parental psychological control (i.e., parents whose behaviors intrude upon their children’s independent thoughts and beliefs) and child anxiety. For example, an increasing correlation between parental psychological control and child anxiety severity has been shown as children transition into adolescence [20, 40, 44]. Furthermore, stronger pooled effect sizes have been demonstrated between parent psychological control and child anxiety in youth ages 13–15 (as opposed to youth ages 10–12 and 16–20) in at least one meta-analysis [40]. Though parental psychological control is a distinct construct from parental accommodation, these findings from previous studies are largely in line with our results suggesting the strongest relationship between parental accommodation and OCS in adolescence (i.e., M = 15 years). This suggests that youth may be particularly vulnerable to effects of parental overinvolvement in beliefs and routines as they transition from childhood into adolescence.

It is also possible that, more broadly, parental accommodation can be less detrimental for younger children’s OCS simply because younger children’s routines and behavior are more closely linked with their parents’ routines than that of an adolescent. Non-familial influences are limited in early/middle-aged children whose day-to-day schedule revolves heavily around their parents, as opposed to adolescents who engage in more autonomous behavior (e.g., choosing their own extracurricular activities, driving themselves, selecting their own peers/romantic partners; [20, 40]). Parental accommodation may be less disruptive to a child’s day when a parent–child routine is more closely linked. Consequently, it is possible that more internal and external conflict occurs when parents try to accommodate their teenager’s behavior to the same degree. This may lead to a worsening of OCS for a variety of reasons (e.g., worsening symptoms are a byproduct of family conflict, accommodation is logistically harder and less effective at reducing short-term distress, but youth still rely on it, etc.).

### Gender as a Moderator

Girls’ anxiety symptoms are more heavily influenced by a parent’s own anxiety or their controlling/over-involved parenting practices [3, 31, 40]. This prior research guided our hypothesis that a similar relationship would exist with respect to the relationship between parental accommodation and OCS. Contrary to our prediction, we found that greater parental accommodation significantly predicted higher OCS in boys, but not in girls. One potential explanation for this contrary finding is that the experience of youths with anxiety does not extend to OCS. There is a notable and well-established increase in girls’ anxiety symptoms (as compared to boys’) during puberty, but the relationship between OCS and puberty is less clear cut [4]. Gender and parenting practices may interact differently with OCS than they do with anxiety symptoms.

Approximately 90% of the parent/guardian respondents within the current study were female (e.g., biological or adoptive mother). This imbalance is not uncommon in parent–child dyad research [48] but may nevertheless impact results. In general, mothers are more likely to accommodate or be involved in their children’s OCD-related rituals than fathers [28, 32]. There may also be gender differences both in the way mothers accommodate their children, and in the way mothers perceive and report their children’s OCS. More discrepancy exists between mother-son reports of symptoms than father-son reports of symptoms [19]. It is possible that mothers may misreport (or in this case, over-report) their sons’ OCS, particularly if they are more involved in compulsions (i.e., more accommodation). More research is needed to elucidate this specific relationship in the context of parental accommodation.

Finally, it is possible that gender differences exist regarding the types of OCS a child displays, or in the way children respond to accommodation or the lack thereof. This may subsequently impact the accommodation–symptom severity relationship and cycle. In an attempt to understand this unexpected gender-related finding, we conducted exploratory post-hoc *t*-tests to evaluate whether gender differences exist with respect to individual items on the FASA. Results indicated a significant gender difference on the item “Has your child become distressed when you have not provided assistance?” such that parents rated boys’ distress as greater than girls. Boys might exhibit more noticeable distress or externalizing behaviors when their parents do not accommodate, which in turn further reinforces accommodation and strengthens the relationship between accommodation and OCS. These findings are in line with the well-established notion that boys are often perceived as more aggressive than girls [8].

Our data did not allow for a gender comparison between types of OCS, but it is possible that this could have played a role as well (e.g., if boys have more compulsions that are disruptive to the family or seen as more disruptive than girls, accommodation may occur more frequently and more potently impact parent report of symptom severity). The role of gender in OCS presentation is not well understood, particularly in research with children, but some gender differences exist with respect to the types of obsessions and compulsions that persist [26]. Replication and extension of these findings are needed to clarify the relationship between gender, parental accommodation, and OCS. Importantly, our sample did not include any individuals who identified as non-binary or gender non-conforming. Future research should take into account differences between biological sex and gender identity when discussing sex or gender-related differences.

## Limitations

A primary limitation extending across both the age and gender moderation analyses is the relatively small sample size, particularly given the multiplicative effect of moderation. This is, however, less of an issue given our use of the PROCESS Macro that includes bootstrapping techniques [18]. Notably, the cross-sectional nature of this research design limits the strength of the findings. An important next step in this line of work, particularly with respect to our age-related findings, will be to longitudinally evaluate how age and gender influence the relationship between accommodation and OCS.

It is also important to note that, while the focus of the present work was OCS, not all participants had an OCD or psychiatric diagnosis. Although the majority of accommodation research to date has examined accommodation in fully clinical samples, we would expect the majority of parents to accommodate their child’s anxiety and related behaviors to some degree, even though the level of accommodation may differ significantly between healthy controls and those with a diagnosis. Future research should consider extending this work within a full sample of youth with an OCD diagnosis. Finally, while our primary outcome measure (SCAS-P: OCD Subscale) is a well-validated measure of OCS, more comprehensive measures exist (e.g., CY-BOCS) that may allow for more in-depth comparison of age- or gender-related differences in youths’ symptoms. Given that previous research has not explored these variables in the manner described herein, we view the present work, using the SCAS-P: OCD, as an appropriate and promising starting point for future inquiry. For example, future research may consider using different measures of OCS such as the CY-BOCS, incorporating multiple measures of OCD-related symptom severity, or OCD-specific measures of parental accommodation within a clinically ascertained sample of youth with an OCD diagnosis.

## Data Availability

No datasets were generated or analysed during the current study.
